# Mediators of repeat mammography in two tailored interventions for Iranian women

**DOI:** 10.1186/s12889-016-2808-4

**Published:** 2016-02-13

**Authors:** Fariba Farhadifar, Yamile Molina, Parvaneh Taymoori, Setareh Akhavan

**Affiliations:** 1 Social Determinants of Health Research Center, Kurdistan University of Medical Sciences, Sanandaj, Iran; 2 Community Health Sciences, School Of Public Health, University of Illinois-Chicago, Chicago, USA; 3 Tehran University of Medical Sciences, Imam Khomini Complex Hospital, Valiasr Hospital, Gynecology Oncology Ward, Tehran, Iran

**Keywords:** Repeat Mammography, Mediators, Intervention, Iranian Women, Breast Cancer

## Abstract

**Background:**

Many theory-based interventions exist that incorporate theoretical constructs (e.g., self-efficacy, behavioral control) believed to increase the likelihood of mammography. Nonetheless, little work to date has examined if increased screening among women receiving such interventions occurs due to changes in these targeted constructs. The aim of this study is to address this gap in the literature in the context of two interventions for improving regular screening among Iranian women.

**Methods:**

A sample of 176 women over 50 years old in Tehran, Iran were randomly allocated into one of these three conditions: 1) an intervention based on Health Belief Model (HBM); 2) an intervention based on an integration of the HBM and selected constructs from the TPB (TPB); and 3) a control group (CON). Questionnaires were administered before the intervention and after a 6-month follow-up. The Preacher and Hayes method of mediation was used in analytic models.

**Results:**

Changes in susceptibility, self-efficacy, and perceived control appeared to mediate HBM-CON differences in screening. Barriers attenuated the mediating effect of self-efficacy. Changes in barriers and self-efficacy appeared to mediate TPB-CON differences in screening.

**Conclusion:**

This study was successful in identifying which theory-based constructs appear to underlie the effectiveness of HBM- and TPB-based interventions. Specific constructs have been identified that should be targeted in clinical practice to increase mammography practices among Iranian women.

## Background

Mammography screening has received scrutiny in the past few years, due to its false positive rates and high rates of indolent breast cancer diagnoses [1–3]. Simultaneously, regular screening remains the most evidence-based screening tool for women with average breast cancer risk [4]. Regular mammography is associated with an approximate 20 % reduction in breast cancer mortality [5]. The majority of literature has focused on women living in developed countries, but early breast cancer detection is also an important challenge for women residing in countries such as Iran [6, 7]. In 2007, 72 % of Iranian breast cancer patients were diagnosed with tumors larger than 2 cm and 63 % had lymph node involvement [8]. Further, breast cancer mortality rates appear to be increasing in Iran [9]. While there are currently no official screening guidelines within Iran, one cost-effectiveness study [10] recommended a national adoption of US Preventive Services Task Force (USPSTF) guidelines [11]. National adoption of these guidelines and implementation of screening programs may have a major public health impact, as alarmingly low screening rates may underlie the disproportionate amount of late stage breast cancer diagnoses and breast cancer-related deaths Iran faces. Indeed, two studies estimated lifetime screening rates among Iranian women to be between 3 % and 12 % [12]. Another study indicated that lifetime screening is not the only public health priority-regular screening rates among Iranian women appears to be as low as 5.7 % [13]. The current study thus focuses on two interventions designed to improve Iranian women’s regular screening, based on USPSTF guidelines (attainment of mammograms every two years for 50–64 year old women).

Successful implementation of programs that promote optimal, guideline-concordant screening requires careful consideration [14]. In the last decade, the importance placed on theory when planning interventions has increased [15, 16]. The majority of interventions in Iran have incorporated the tenets of the Health Belief Model (HBM), which provides important information concerning intrapersonal factors associated with health behavior [16, 17]. In the context of breast cancer, this theory conceptualizes mammography screening to be affected by the following factors: (1) perceived susceptibility or risk of being diagnosed with breast cancer; (2) perceived severity or seriousness about breast cancer (e.g., consequences of a diagnosis) (3) perceived benefits or effectiveness of mammography to detect breast cancer; (4) perceived barriers to obtain a mammogram (e.g., transportation, childcare); and (5) self-efficacy or perceived ability to accomplish the behavior successfully [18]. Many HBM interventions involve patient counseling in group-and individual settings, wherein patient-staff interactions are focused on these specific factors. For example, HBM interventions designed to improve self-efficacy may involve research staff encouraging women to reflect on their ability to obtain mammograms.

Another useful framework to guide mammography screening interventions is the Theory of Planned Behavior (TPB), which addresses the intrapersonal factors described above as well as the perception of environmental factors (e.g., contextual, interpersonal) [18]. In part, these interventions may work on perceptions through actualizing positive contextual factors and interpersonal experiences. For example, the TPB addresses subjective norms [19, 20] and the perception of contextual factors or perceived behavioral control [21], both of which have been tied to mammography screening [19, 21]. Subjective norms are conceptualized as perception that screening is considered appropriate and important by one’s broader society and social networks, including family and friends [22–24]. Women may perceive a greater amount of sociocultural approval about screening if they experience interpersonal interactions wherein others suggest they obtain mammograms [25]. Thus, interventions may increase subjective norms through increasing recommendations by family, friends, and other women to obtain mammograms. Perceived control is defined as women’s perceived ability to obtain a mammogram in the face of contextual barriers [21]. For example, in a country with no national mammography program, women with a greater amount of perceived control may be more likely to identify private and non-profit resources available to obtain a mammogram than women with low perceived control [26]. TPB interventions may target perceived behavioral control through counseling patients (e.g., initiating conversations with providers) as well as facilitating connections between patients and resources (e.g., increasing access to provider referrals).

Notably, perceived control and self-efficacy overlap conceptually, wherein both concern women’s perceptions about their abilities to behave in a certain manner. Some scholars have indicated that perceived behavioral control and self-efficacy are not conceptually different constructs [27]. In contrast, others [28, 29] have defined self-efficacy as more holistic than perceived control. Specifically, self-efficacy can be defined as women’s perceived ability to accomplish a behavior in the face of different, simultaneous barriers, including intrapersonal (e.g., fear) and environmental factors (e.g., healthcare access, social stigma). Perceived control, conversely, can be defined as women’s perceived ability to accomplish a behavior relative to environmental factors only.

Researchers frequently refer to a theoretical framework (e.g. HBM or TPB) when discussing intervention development and evaluation. Nonetheless, few studies have conducted mediation analyses to identify if increased screening among women receiving these interventions is a result of changes in targeted theoretical constructs. Such information would identify the specific mechanisms underlying intervention efficacy. This identification would optimize future adaptation and allow for more efficient interventions and clinical programs.

We sought to address this gap in the literature by examining which targeted mechanisms underlie the increased uptake of regular screening among nonadherent Iranian women participating in two 6-month tailored interventions (HBM, TPB) relative to Iranian women participating in a control group (CON). Both interventions included group and individualized components. Nonetheless, there were significant differences with regard to which stakeholders were involved. The HBM intervention used a patient counseling approach, such that participants engaged with research staff. The TPB intervention used a multi-faceted approach, such that participants engaged with research staff as well as family, friends, peer participants, and healthcare providers. Notably, family, friends, and healthcare providers also engaged with staff to optimize women’s actual experiences with positive interpersonal and contextual factors. This quasi-experimental study was based in the city of Tehran and similar to our previous randomized controlled trial in the city of Sanandaj [30]. We hypothesized that: 1) study arm differences in regular screening between HBM and CON groups would be a result of greater changes in HBM theoretical constructs (susceptibility, severity, benefits, barriers, self-efficacy); and 2) study arm differences in regular screening between TPB and CON groups would be a result of greater changes in HBM (susceptibility, severity, benefits, barriers, self-efficacy) and TPB theoretical constructs (subjective norms, perceived control).

## Methods

The research described below was approved by the Tehran University ethics board and the appropriate educational authorities.

### Study design and setting

The current study was based in Tehran. As described above, this trial was an adaptation of our previous work based in the city of Sanandaj [30]. Key differences between these studies include the use of multiple healthcare centers per study arm within Sanandaj and only one center per study arm in Tehran (3 total). This decision was due to previous research concerning Tehrani women’s social network characteristics [31]. Thus, we randomly assigned study arms across the three centers to minimize contamination effects, using a table of random numbers [32].

### Participant recruitment and retention

Eligibility criteria for the current study were: 1) age of 50–64 years old; 2) no history of breast cancer; 3) lifetime history of mammography use; 4) a lack of obtaining a mammogram within the past 2–3 years; 4) no intention of obtaining a mammogram within the next year; and 5) an ability to read and write. To determine the sample size needed to test our hypotheses, we used a power calculation based on Cohen, Cohen, West, and Aiken’s [33] $n=\frac{L}{f^2}+k+1$, where k is the number of predictors/mediators, f is an effect size, and L is a tabled value corresponding to a specific power value [33]. The minimum required sample size to detect a medium effect size (0.26), given seven predictors/mediators, an alpha of .05 and a power level of .80, was 125. We contacted 185 women, assuming we would obtain a minimum of 125 women who were eligible, agreed to participate and completed the trial entirely. Between 61 and 62 women between 50–64 years old were then randomly selected from each clinic using a table of random numbers. Staff contacted women by phone or through a home visit, if their phone number not available.

Of the 185 contacted, 6 women were eligible, but refused to participate in the trial. The remaining 179 women were eligible and consented. Prior to beginning in their clinic’s program (HBM, TPB, CON), participants received a written information sheet and consent form to sign. Of the 179 eligible women who consented and began the study, 3 did not complete the study (2 in HBM group, 1 in CON group). The current study focuses on the 176 eligible women who were consented and completed the study (TPB = 62; HBM = 58; CON = 56). After the study ended, participants were given $10 for their time and effort.

### Intervention development and procedures

Table 1 details the 20-week timeline, targeted constructs, and educational methods used for HBM and TPB arms. Women in the CON group did not interact with staff throughout weeks 1–20. Nonetheless, after Week 20, all participants, including women in the CON group, were sent reminders and pamphlets concerning the importance of mammography.

**Table 1:** Breakdown of specific intervention components by time, targeted construct and methods

*Time and groups receiving session*	*Theory*	*Targeted theoretical constructs*	*Methods used*
*Week 1*–*6*: *HBM*, *TPB*	*HBM*	*Perceived threat of breast cancer*	*Lecture for group educational session targeting*: -Facts about breast cancer -Risk factors for breast cancer -Effective messages to increase the perceived threat of breast cancer and high levels of response efficacy of mammography.
	*HBM*	*Perceived benefits to mammography*	*Lecture for group educational session targeting*: -Facts about recommended rescreening frequency and its importance -Benefits of mammography for early detection and thus a higher chance of survival Summary pamphlet on the benefits of mammography.
*Week 8*–*9*: *HBM*, *TPB*	*HBM*	*Perceived barriers to mammography*	*Lecture for group educational session targeting*: -Personal and environmental barriers -Broad information about how to address barriers (e.g., low-cost mammography exists) *Tailored individual counseling and reminder cards*/ *pamphlets targeting*: -Women’s specific barriers (e.g., how to obtain a mammogram in relation to women’s specific spatial and temporal constraints) -Women’s response efficacy of the mammography process and perceptions about the benefits to mammography
*Week10* –*13*: *HBM*, *TPB*	*HBM*	*Perceived self*-*efficacy*	*Lecture for group educational session targeting*: -The ability to schedule physician visits and logistical barriers for mammography appointments -The ability to address psychosocial barriers -Women’s ability to act and survive if they receive a diagnosis (e.g., immediate treatment initiation) *Tailored individual counseling and reminder cards*/*pamphlets targeting*: -Women’s knowledge about their yearly schedule to obtain mammograms -Women’s definite action plans about how they can address matters of cost, time, or transportation (e.g., how to identify locations that offer reduced-price mammograms) -Women’s ability to talk to providers about their specific psychosocial concerns about mammography (e.g., fear of a diagnosis)
*Week 14* – *17*: *TPB*	*TPB*	*Perceived subjective norms*, *perceived behavioral control* (*only for TPB* *group*)	*Lecture for group educational session with participants emphasizing*: -Peer support, exposure modeling and interpersonal norms. -Skills to develop social network by sharing commitment and plans for obtaining mammograms -Participants’ beliefs to accept that it is up to them to use breast cancer screening behaviors in different environmental conditions -Participants’ skills in engaging healthcare providers about mammography referrals *Tailored individual counseling and reminder cards*/*pamphlets targeting*: -Identification of family and friends who can support women in obtaining mammograms -Participants’ skills in engaging healthcare providers about mammography referrals *Small group educational session with participants*’ *family and friends targeting*: -Family/friends’ skills and abilities to support women in their ability to obtain mammograms
*Week 18*: *TPB*	*TPB*	*Perceived control* (*only for TPB group*)	*Tailored individual counseling and reminders*/*pamphlets targeting*: -Women’s awareness of information regarding scheduling mammography appointments and providers with whom they can engage about appointments -Women’s specific experiences and unique challenges with environmental factors (e.g., how to minimize fear and social stigma by having a friend attend one’s appointment)
*Week 20*: *HBM*, *TPB*	*HBM* *TPB*	(Self-efficacy - only for HBM group) (Perceived behavioral control - only for TPB group)	*Tailored individual counseling targeting*: -Women’s self-identified goals and encouragement through positive feedback and verbal persuasion concerning unique intrapersonal barriers -Women’s self-identified unique environmental barriers to attaining mammograms and strategies concerning how to respond to environmental challenges

*Abbreviations: HBM* intervention based on the health belief model, *TPB* intervention based on the health belief and the theory of planned behavior

During the first thirteen weeks (weeks 1–13), women in HBM and TPB arms participated in HBM-oriented group and individual counseling sessions focused on the perceived threat of breast cancer (i.e., perceived susceptibility/seriousness), the benefits of obtaining mammograms, the barriers to obtaining mammograms, and self-efficacy. Group sessions lasted 45–60 minutes. Participants then received a 10–15 minute individual counseling session, based on their baseline questionnaire responses and reactions during group sessions. Specifically, baseline questionnaire sand group sessions allowed staff to identify which barriers each participant had (e.g., lack of knowledge about what a mammogram is, embarrassment). Staff then addressed these barriers during individual counseling sessions.

During weeks 14–18, women in the HBM arm did not interact with staff. Women in the TPB group however received four group and individual counseling sessions focused on subjective norms and perceived behavioral control during those weeks. These sessions differed from HBM sessions, wherein staff not only engaged participants, but also facilitated participants’ interactions with peers and their larger social networks with regard to mammography. For example, during subjective norms sessions, small groups were formed to promote peer support and increase exposure to positive subjective norms concerning mammography use. Next, participants were asked during individual counseling sessions to identify and provide contact information for five important relatives they thought might help remind them about scheduling a mammogram. Research staff subsequently contacted relatives and invited them to participate in group sessions that focused on different ways to talk to women about mammography use, including short phone messages and reminders about the need to obtain a mammogram when delivering birthday, wedding anniversary, and mother/woman’s day gifts. Regarding perceived behavioral control, women were trained as to how to resolve environmental challenges through interpersonal interactions, including engaging physicians to obtain screening referrals. Relatedly, during the 18^th^ week of the intervention, TPB participants received signed reminder messages by a gynecologist physician, who had interacted with research staff, regarding scheduling mammography appointments.

During Week 20, women in the HBM group participated in individualized counseling sessions about their previously identified goals from Weeks 1–13 and their strategies for addressing their specific barriers. These counseling sessions thus targeted self-efficacy through reminding women of their plans to obtain mammograms and providing positive feedback about these plans [18]. Women in the TPB group also participated in individualized counseling sessions. These sessions however focused on perceived behavioral control. Specifically, women were asked to consider their unique environmental challenges (e.g., lack of provider referral, limited operating hours for mammography centers) as well as their own potential cognitive and behavioral strategies for responding to these challenges.

### Instruments

All participants (CON, HBM, TPB) interacted twice with research staff to complete surveys at before and six months after the 20-week intervention period.

#### Socio-demographic and clinical variables

Baseline and follow-up questionnaires included the following variables: age, marital status, employment status, educational level, family history of breast cancer, and health insurance.

#### Theoretical constructs

Seven theoretical constructs were assessed as potential mediators in this study: susceptibility, severity, benefits, barriers, self-efficacy, perceived control and subjective norms. For all measures, we used a standard forward-backward translation technique (English-Farsi; Farsi-English) to translate the instrument into Farsi [34], using two professional, bilingual translators. Three gynecologists, two health education professors, one psychologist, and two public nursing professors reviewed instruments to ensure that it could be understood by Iranian women and in the most appropriate terms.

HBM measures were based on Champion’s revised Health Belief Model Scale (CHBMS) [35–37] and self-efficacy instruments [38]. The validity and reliability of our measures (Cronbach’s alphas ≥ 0.70) were previously demonstrated in Sanandaj [39]. Based on these analysis, we administered 3-item perceived susceptibility of breast cancer (Cronbach’s alpha = 0.85), 7-item perceived severity of breast cancer (Cronbach’s alpha = 0.80), 6-item perceived mammography benefits (Cronbach’s alpha = 0.77), 9-item perceived mammography barriers (Cronbach’s alpha = 0.74), and 10-item self-efficacy instrument (e.g., “I can arrange transportation to get a mammogram”; Cronbach’s alpha = 0.87). Items were rated on a 4-point Likert scale. For all subscales except for self-efficacy, the response anchors were strongly disagree to strongly agree. For self-efficacy, the anchors were not at all confident to very confident.

TPB measures were single items adapted from an existing mammography perceived control and subjective norms scales [28]. These items were also previously validated in Sanandaj [39]. Subjective norms were assessed with the following item: “Most of the people who are significant to you expect that you must get a mammogram when you are due” on a 4-point scale ranging from 1 (strongly disagree) through 4 (strongly agree). The test-retest reliability coefficient for this item was 0.81. Perceived control was assessed with the following statement: “How much control do you have, over whether you get a mammogram when you are due?.” The item used a 4-point response scale: 1 = not under my control to 4 = under my control. The test-retest reliability coefficient for this item was 0.85.

For all variables, we first calculated the average of items at different time points. Change scores were then calculated for all of these variables as the difference between follow-up and baseline summary scores.

#### Mammography screening

As described above, women provided self-report data concerning their lifetime screening history when undergoing eligibility screening for this trial (yes/no). Thus, all participants self-reported attainment of at least one mammogram within their lifetime. To be eligible for the trial, they also should not have obtained a mammogram within the past 2–3 years (yes/no). With regard to mammography screening six months after the intervention, medical record data were used for women who agreed to abstraction (78 %). For those who did not agree to abstraction, self-report data were used (22 %). The variable was categorized into 0 = Did Not Obtain A Mammogram and 1 = Obtained A Mammogram.

### Data analysis

We first assessed study arm differences in socio-demographic, clinical, and mammography screening variables through bivariate analyses (chi-square tests, analyses of variance). We then assessed the relationships between theoretical constructs through Pearson’s correlations.

We next assessed the ‘a’ and ‘b’ paths of theorized mediation pathways. To test the ‘a’ paths (differences in potential mediators by predictor groups), we conducted multivariate analysis of co-variance (MANCOVA) to assess differences in HBM and TPB theoretical constructs across study arms, after adjusting for socio-demographic and clinical covariates. We chose MANCOVA, as it is a useful, simplistic test for variables that are conceptually and empirically related [30]. If omnibus tests were significant, we conducted post-hoc analyses to assess differences in constructs between women in the control group relative to women in intervention arms. To test the ‘b’ path (examining how potential mediators are related to outcomes), we conducted a multivariable logistic regression model, including all study arms, to examine which HBM and TPB theoretical constructs were associated with screening six months after the intervention. We then conducted sensitivity analyses, wherein we conducted logistic regression models 1) only for HBM and CON participants; and 2) only for HBM and TPB participants. Potential mediator variables were identified as HBM and TPB theoretical constructs which differed across intervention and control arms (“a path”) and were associated with screening (“b path”). For example, if HBM and CON groups differed with regard to perceived barriers and perceived barriers were associated with screening, then perceived barriers was tested as a mediator. If, however, TPB and CON groups did not differ with regard to perceived barrier, we did not include it as a mediator, despite its association with mammography screening.

We finally conducted mediation models using the Preacher and Hayes method, which is particularly useful for testing multiple, potentially interrelated, mediators [40]. This bootstrap method is a nonparametric procedure that involves re-sampling from the data set multiple times (5,000 for this study) and generating a sampling distribution. We calculated 95 % confidence intervals of the effect of being in an intervention on mammography screening through the mediators described above. Both interventions (HBM and TPB) were compared to the control group in separate analyses to assess the mediation effects. We determined percentage mediated as a function of the indirect pathway (A*B or the product of intervention group on changed theoretical constructs and theoretical constructs on screening) divided by the sum of the direct effect (intervention/control group on screening) and the indirect effect (A*B).

## Results

Table 2 depicts socio-demographic, clinical, and screening variables by study arms. The majority of socio-demographic and clinical variables did not vary across study arms. There were however significant differences in marital status, χ^2^ (2) = 22.50, *p* < .001. We therefore included marital status as a covariate in all subsequent analyses. Notably, women in the HBM (64 %) and TPB arms (60 %) were significantly more likely to obtain a mammogram relative to women in the CON arm (22 %; χ^2^ (2) = 24.17, *p* < .0001). Significant relationships existed for all bivariate relationships between theoretical constructs. Absolute values ranged between 0.25 (subjective norms and benefits) and 0.67 (self-efficacy and benefits).

**Table 2:** Socio-demographic and clinical variables by study arm

	CON (*n* = 56)	HBM (*n* = 58)	TPB (*n* = 62)	Total (*n* = 176)	*p*-*value*
	*M* (*SD*)	*M* (*SD*)	*M* (*SD*)	*M* (*SD*)	
Age	58.34 (4.99)	60. 26 (5.69)	58.85 (5.62)	59.15 (5.49)	0.15
	*N* (%)	*N* (%)	*N* (%)	*N* (%)	
<High school	10 (18)	8 (14)	16 (26)	34 (19)	0.41
Good/very good income	39 (70)	33 (57)	41 (66)	113 (64)	0.33
Employed	25 (45)	33 (45)	33 (53)	84 (48)	0.56
Married	25 (45)	50 (86)	43 (69)	118 (67)	0.001
Insured	51 (91)	52 (89)	46 (79)	149 (85)	0.21
% any breast problem	6 (11)	4 (7)	10 (6)	20 (11)	0.26
Family history of breast cancer	3 (5)	1 (2)	5 (8)	9 (5)	0.28
Obtained mammogram	12 (22)	36 (62)	40 (65)	88 (50)	<0.0001

*Abbreviations: CON* control group, *HBM* intervention based on the health belief model, *TPB* intervention based on the health belief and theory of planned behavior, *n* (%) number and percent of women who reported obtaining a mammogram within six months following the intervention

### Study arm differences in theoretical constructs

Study arm differences in theoretical constructs are depicted in Table 3, after adjusting for marital status. Women in the HBM arm had greater gains in all factors relative to women in the CON arm, except for barriers, for which they experienced a greater reduction. Relative to women in the CON arm, women in the TPB arm experienced greater gains in self-efficacy, perceived control, and subjective norms as well as a greater reduction in barriers.

**Table 3:** Specific values for pretest, posttest, and change scores in theoretical constructs across intervention and control groups, adjusted for marital status

Hypothesized mediators	*Baseline*			*Six months follow*-*up*			*Change scores* (*Post*-*intervention* – *Pre*-*intervention*)				
	*CON* (*n* = *56*)	*HBM* (*n* = *58*)	*TPB* (*n* = *62*)	*CON* (*n* = *56*)	*HBM* (*n* = *58*)	*TPB* (*n* = *62*)	*CON* (*n* = *56*)	*HBM* (*n* = *58*)	*CON*-*HBM p*-*value*	*TPB* (*n* = *62*)	*CON*-*TPB p*-*value*
Perceived susceptibility	3.07 (0.13)	3.36 (0.13)	3.32 (0.12)	3.17 (0.15)	4.21 (0.15)	3.62 (0.14)	0.12 (0.19)	0.85 (0.18)	0.006	0.31 (0.17)	0.42
Perceived severity	2.71 (0.13)	3.03 (0.13)	2.91 (0.12)	2.77 (0.14)	3.92 (0.13)	3.23 (0.13)	0.06 (0.19)	0.89 (0.18)	0.002	0.41 (0.17)	0.16
Perceived benefits	2.74 (0.09)	2.55 (0.08)	2.79 (0.08)	3.27 (0.09)	3.57 (0.08)	3.25 (0.08)	0.53 (0.12)	1.03 (0.12)	0.005	0.46 (0.11)	0.65
Perceived barriers	2.84 (0.08)	2.96 (0.07)	2.73 (0.07)	2.24 (0.08)	1.79 (0.07)	1.83 (0.07)	−0.50 (0.10)	−1.17 (0.10)	<0.0001	−0.91 (0.10)	0.03
Perceived self-efficacy	1.47 (0.06)	1.45 (0.061)	1.48 (0.06)	1.80 (0.10)	2.62 (0.10)	2.38 (0.09)	0.34 (0.11)	1.12 (0.11)	<0.0001	0.91 (0.10)	<0.0001
Perceived behavioral control	1.63 (0.06)	1.60 (0.06)	1.61 (0.05)	1.82 (0.07)	2.21 (0.07)	2.34 (0.06)	0.18 (0.08)	0.62 (0.08)	<0.0001	0.73 (0.08)	<0.0001
Subjective norms	2.93 (0.12)	2.76 (0.11)	2.84 (0.11)	3.03 (0.09)	3.37 (0.08)	3.66 (0.08)	0.10 (0.13)	0.61 (0.13)	0.009	0.82 (0.12)	<0.0001

*Abbreviations: CON* control group, *HBM* intervention based on the health belief model, *TPB* intervention based on the health belief and theory of planned behavior

### Theoretical constructs and screening

We examined if changes in targeted theoretical constructs in relation to screening across study arms, after adjusting for marital status. Findings suggested that increased susceptibility, self-efficacy, and control were associated with increased odds of obtaining a mammogram. Conversely, increased barriers were associated with decreased odds of obtaining a mammogram (Table 4). When including only HBM and CON participants, odds of obtaining a mammogram was associated with changes in susceptibility, self-efficacy, and perceived control. When including only TPB and CON participants, odds of obtaining a mammogram was associated with changes in barriers and self-efficacy. Perceived control was also marginally associated with odds of obtaining a mammogram.

**Table 4:** Predictors of mammography screening, after adjusting for marital status

	*Entire sample* (*n* = *176*)		*HBM & CON only* (*n* = *114*)		*TPB & CON only* (*n* = *118*)	
	*Adjusted OR (95 % CI)*	*p*-*value*	*Adjusted OR (95 % CI)*	*p*-*value*	*Adjusted OR (95 % CI)*	*p*-*value*
Perceived susceptibility	1.7 (1.1, 2.6)	0.01	1.9 (1.1, 3.1)	0.02	1.6 (0.9, 3.0)	0.13
Perceived severity	1.0 (0.7, 1.6)	0.88	0.8 (0.5, 1.2)	0.28	1.0 (0.6, 1.7)	0.96
Perceived benefits	0.7 (0.4, 1.3)	0.280.	1.3 (0.5, 3.3)	0.56	1.8 (0.7, 4.8)	0.26
Perceived barriers	0.4 (0.2, 0.8)	0.005	0.8 (0.3, 2.1)	0.61	0.3 (0.1, 0.7)	0.008
Perceived self-efficacy	2.0 (1.1, 3.8)	0.04	2.9 (1.2, 7.3)	0.02	13.0 (3.4, 49.2)	<0.0001
Perceived behavioral control	2.5 (1.1, 5.3)	0.02	4.6 (1.5, 13.7)	0.007	2.9 (0.9, 9.6)	0.08
Subjective norms	1.0 (0.6, 1.5)	0.92	1.0 (0.5, 2.1)	0.92	0.7 (0.3, 1.6)	0.43

*Abbreviations: 95 % CI* confident intervals

### Mediation models

**Fig. 1:**
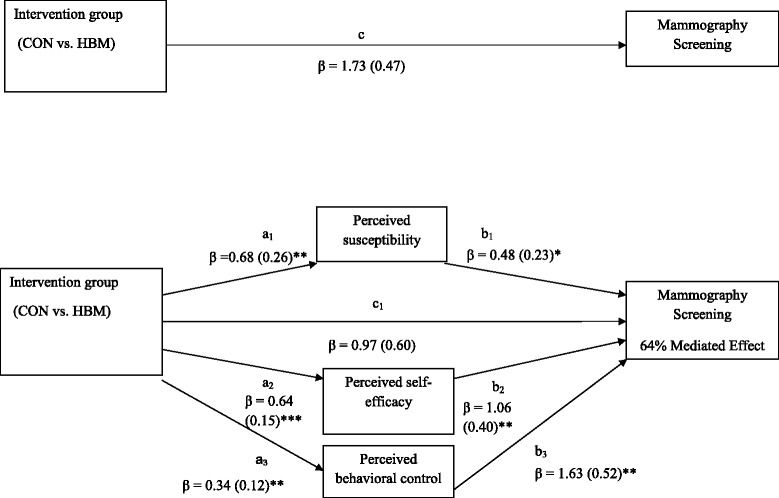
Multiple mediation model of the relationship of intervention group status (CON vs. HBM) and mammography screening. Marital status was included as a covariate. All coefficients represent unstandardized regression coefficients with standard errors in parentheses. Mediated effect is calculated as a function of the indirect pathway (A*B or the product of intervention group on changed theoretical constructs and theoretical constructs on screening) divided by the sum of the direct effect (intervention/control group and screening) and the indirect effect (A*B). *p < .05 **p < .01 ***p < .0001

**Fig. 2:**
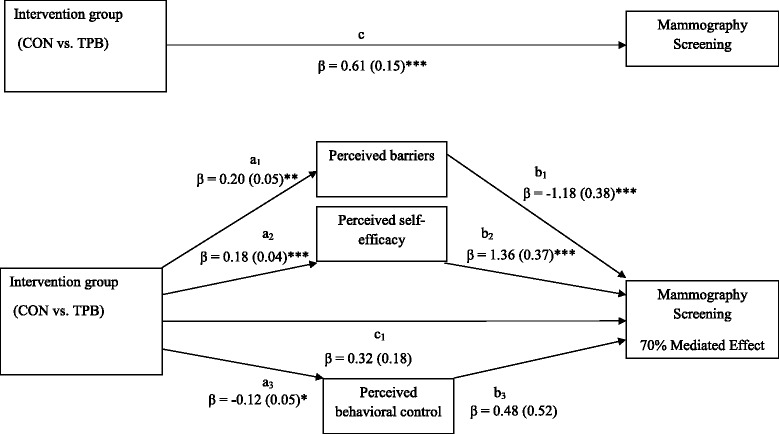
Multiple mediation model of the relationship of intervention group status (CON vs. TPB) and mammography screening. Marital status was included as a covariate. All coefficients represent unstandardized regression coefficients with standard errors in parentheses. Mediated effect is calculated as a function of the indirect pathway (A*B or the product of intervention group on changed theoretical constructs and theoretical constructs on screening) divided by the sum of the direct effect (intervention/control group and screening) and the indirect effect (A*B). *p < .05 **p < .01 ***p < .0001

Given these findings, we first tested the mediating roles of susceptibility, self-efficacy, and perceived control (absolute intercorrelation coefficient values: 0.29-0.59) in HBM and CON differences in mammography screening. Findings from the first model suggested a full mediation of differences in mammography screening between HBM and CON arms (64 % mediated; Fig. 1). Second, we tested the mediating roles of barriers, self-efficacy, and perceived control (absolute intercorrelation coefficient values: 0.45–0.53) in TPB and CON differences in mammography screening. This model also suggested full mediation and appeared to be driven by study arm differences in changes in barriers and self-efficacy (70 % mediated; Fig. 2).

We conducted sensitivity analyses, given preliminary findings suggested strong associations between theoretical constructs. To provide comparable models with the same theoretical constructs, we included barriers as another mediator when examining HBM-CON differences and susceptibility as another mediator when examining HBM-TPB differences. With regard to the HBM-CON model, susceptibility and perceived control emerged as significant mediators. Barriers and self-efficacy, however, were not significant mediators. This finding suggests that perceived barriers may positively confound the mediating effect of perceived self-efficacy within the context of HBM-CON differences in mammography screening. With regard to the TPB-CON model, similar patterns emerged. Barriers and self-efficacy emerged as significant mediators, whereas susceptibility and perceived control did not.

## Discussion

The current study is among the first to examine if the effectiveness of theory-based interventions in improving mammography screening is due to changes in targeted constructs. Women in the HBM-based group appeared to have increased screening relative to women in the CON group due to greater susceptibility, self-efficacy, and perceived control. Nonetheless, barriers appeared to be an underlying confounder in the mediating effect of self-efficacy for this set of study arm differences. Women in the TPB-based group appeared to have greater odds of obtaining mammograms relative to women in the CON group due to greater reductions in barriers and increased self-efficacy. These preliminary findings have important implications concerning which constructs should be targeted in future HBM- and TPB-based interventions in Iran. They further provide a theoretical platform by which other theory-based interventions may be evaluated.

Similar to our previous work in another city of Iran [30], the current study found that women participating in intervention arms experienced significant changes in targeted theoretical constructs. There were however some differences. Within the Sanandaj study, women across both intervention arms experienced significantly greater increases in susceptibility, severity, benefits, subjective norms, and perceived control as well as a significant reduction in barriers relative to women in the control group. Within the current study, the HBM intervention appeared to work similarly in terms of significant changes in theoretical constructs relative to women in the control group. Nonetheless, women in the TPB intervention did not experience significant changes with regard to susceptibility, severity, and benefits relative to women in the control group. These differences may be due to differences in study design (e.g., randomized controlled trial versus quasi-experimental design) or actual differences in how these concepts are experienced across distinct regions in Iran. Our findings highlight the importance of replication in order to characterize intervention effectiveness across different populations and to obtain sufficient data to assess mediators and moderators underlying variation in effectiveness.

Across study arms, increased susceptibility, self-efficacy and perceived control was associated with greater odds of obtaining mammograms, after adjusting for marital status. In addition, a reduction in perceived barriers was associated with greater odds of obtaining mammograms. Our work is concurrent with observational studies in Iran and other countries that suggest mammography plans and practices are related to fewer barriers [41–43], greater self-efficacy [44–46], and greater perceived control [30]. Our findings further align with some work suggesting increased susceptibility is associated with greater guideline-concordant mammography screening [47, 48], although other work has indicated that over-elevated susceptibility might be associated with non-adherence [49, 50]. It is further worthwhile to consider that our study did not find benefits, severity, or subjective norms to be associated with mammography screening, contrary to HBM and TPB frameworks. One potential interpretation is that these factors strongly overlap with other theoretical constructs, which may act as confounders or mediators. For example, the relationships of subjective norms and social factors more broadly with mammography screening may be mediated or confounded by self-efficacy and/or perceived control [44].

We found important significant differences in mediators of screening. Specifically, reduced barriers and increased self-efficacy did not consistently mediate screening differences between women in the CON and HBM arms, but did significantly mediate differences between women in the CON and TPB arms. Conversely, changes in susceptibility appeared to mediate differences in screening between CON and HBM arms, but not between CON and TPB arms. Such findings appear to be counterintuitive, given both HBM and TPB arms experienced the same HBM materials, except for Week 20. Simultaneously, perceived control appeared to mediate differences in relation to the HBM-based group, but not the TPB-based group, despite a lack of intervention content that was pertinent to this construct. It is worthwhile to consider that the relationships between the theoretical constructs may confound and mediate not only bivariate associations, but also mediating effects. Our sensitivity analyses revealed such a scenario for self-efficacy as a mediator in the context of HBM-CON differences, wherein inclusion of barriers in the model attenuated its role as a mediator. This may be because barriers serve as mediators or confounders in this three-variable relationship.

Our findings present a complex picture. On the one hand, the current study was motivated by the conceptualization that mediation models can identify the theoretical constructs that serve as mechanisms underlying intervention efficacy. Such models may result in evidence to suggest that interventions can be refined to be shorter and targeted toward fewer theoretical constructs. On the other hand, however, our findings indicate that targeted constructs are not only interconnected, but may serve as confounders and mediators in bivariate and multivariable associations. Specifically, participants’ exposure to intervention materials concerning some constructs may unintentionally result in changes in other constructs as well as the relationship between other constructs and screening. Such findings suggest that interventions’ content should continue to focus on all theoretical constructs, as intervention materials may affect not only the targeted theoretical constructs, but also interrelated theoretical constructs and their associations with screening. An obvious next step is to apply structural equation modeling in order to understand further the matrix of targeted constructs underlying intervention efficacy.

There are a number of limitations that must be noted. First, although this work was longitudinal, assessment of mediators and the outcome of interest occurred at the same time. Given this, causal relationships should not be inferred. Second, our mediation models do not account for all contributing factors to women’s screening practices, but only the constructs that were targeted in the interventions (i.e., intrapersonal and perceived environmental factors). Nonetheless, actual environmental factors likely contribute to the alarmingly low rates of regular screening. Future work should include these factors in analyses and examine their relative contributions within the context of psychosocial interventions that address predominantly intrapersonal and perceived environmental factors. A third limitation concerns the role of measurement for our analyses. Specifically, we used both single- and multiple-item measures, which may have influenced our findings. Our decision to use single-item measures for subjective norms and perceived control was based on the availability of evidence-based scales that measured direct forms of these perceived constructs [1, 18, 28, 29, 44, 51]. Future studies should develop and use multiple-item instruments that directly measure both subjective norms and perceived control to confirm our findings. Another important limitation regards our operationalization of perceived behavioral control. Conceptually, this construct is very similar to self-efficacy, but focuses on environmental and systemic factors [28, 29]. Our single-item instrument did not however directly measure women’s perceived control relative to specific environmental and systemic factors, which may have influenced our findings when including both measures (e.g., potential for multicollinearity). This limited type of operationalization has been discussed by researchers such as Ajzen, who have indicated that these constructs may be the same and differences may be a result of measurement error [27]. Our study thus is not equipped to contribute to this dialogue. Nonetheless, our counterintuitive findings from mediation models within the context of different interventions highlight its importance and the need for rigorous evaluation and comparison of construct validity for these two theoretical constructs. Future studies are warranted that develop and include multiple-item measures that specify women’s perceived control relative to specific constructs (e.g., in the face of no national screening program) in order to confirm our findings. Fourth, TPB topics were continuously presented last, in order to control for order effects across study arms. Ordering effects may have influenced which factors appeared to be the mechanisms underlying intervention success. Future studies should replicate our findings when comparing three adaptations of the HBM + TPB intervention, wherein TPB interventions are presented first and last as well as interspersed. Fifth, women who decided to participate throughout the intervention may have been more motivated to obtain a mammogram than women who were lost to follow-up and women who decided to not participate at all. Sixth, our study is a replication of a previous randomized controlled trial. A major difference in designs concerns the use of multiple healthcare centers and individual-level randomization in the Sanandaj study [30] compared to a quasi-experimental design that administered interventions at three separate hospitals, which may impact the generalizability of this study. Our study findings may further not be applicable in Western societies, other non-Western societies, or even other regions of Iran. Another major limitation concerns our measurement of mammography prior to the trial. In order to be eligible, women had to self-report their utilization of mammography at some point within their past and lack of mammography screening within the past 2–3 years. We did not however collect specific dates and detailed history about the time that had lapsed since the last mammogram. This is a limitation, given mammography history may be a confounder in associations found. Further, these data are self-report, which are often problematic in terms of measurement error [52]. Future trials should confirm our findings, wherein lifetime as well as post-intervention mammography history are assessed based on medical record abstraction. In addition, we focused on repeat mammography utilization; notably, however, women who obtain at least one mammogram are more likely to obtain mammograms in the future [53]. Our intervention may be particularly needed by women with no lifetime history of mammography. Simultaneously, we note that repeat mammography is particularly low in Iran [54]. Thus there is a need for these types of interventions in the context of both mammography behavior initiation and maintenance.

In addition to its limitations as a research study, there are important concerns regarding our results can translate to routine care. There are several potential venues for practitioners to explore. First, future implementation efforts may compare the relative cost effectiveness of relying on in-person group sessions only, phone-based individualized sessions, or the original combined approach. Other options may include other modes of communication, including use of e-mail and the Internet for the individualized components of our program. Another potential way to implement this intervention may be through hospital-based navigators, who can facilitate interactions between women and their providers in a similar fashion as accomplished by our study staff. Indeed, such patient education and counseling while addressing systemic and environmental factors are common components of navigation programs [55, 56].

## Conclusion

Our findings suggest that a reduction of barriers and gains in susceptibility, self-efficacy, and perceived control may be useful toward improving regular screening among Iranian women. This research is important, because it has implications for both future practice and research concerning these different frameworks. Future research should use: 1) longitudinal designs that disentangle the timing of mediators and outcomes; 2) objective measures of actual contextual factors as well as perceived contextual factors; 3) multi-item measures that directly measure and confirm the roles of subjective norms and perceived behavioral control relative to mammography screening; 4) counterbalancing in presentation of HBM and TPB topics; 5) designs that allow for more generalizability (e.g., individually randomized controlled trial, random assignment of multiple healthcare centers); and 6) assessment of intentional and unintentional effects of intervention materials for targeted constructs and other closely related constructs. Future evidence-based practice should consider different routes toward implementation of our findings into routine practice, including different modes of delivery.

## Abbreviations

CHBMS: Champion’s Health Belief Model Scale
CON: control
HBM: Health Belief Model
PCA: principal component analysis
TPB: Theory of Planned Behavior
